# An intelligent listening framework for capturing encounter notes from a doctor-patient dialog

**DOI:** 10.1186/1472-6947-9-S1-S3

**Published:** 2009-11-03

**Authors:** Jeffrey G Klann, Peter Szolovits

**Affiliations:** 1 Medical Informatics, Regenstrief Institute, Indianapolis, IN 46202, USA; 2 Health Informatics, Indiana University, Indianapolis, IN 46202, USA; 3 Clinical Decision Making Group, Computer Science and Artificial Intelligence Laboratory, Massachusetts Institute of Technology, Cambridge, MA 02139, USA

## Abstract

**Background:**

Capturing accurate and machine-interpretable primary data from clinical encounters is a challenging task, yet critical to the integrity of the practice of medicine. We explore the intriguing possibility that technology can help accurately capture structured data from the clinical encounter using a combination of automated speech recognition (ASR) systems and tools for extraction of clinical meaning from narrative medical text. Our goal is to produce a displayed evolving encounter note, visible and editable (using speech) during the encounter.

**Results:**

This is very ambitious, and so far we have taken only the most preliminary steps. We report a simple proof-of-concept system and the design of the more comprehensive one we are building, discussing both the engineering design and challenges encountered. Without a formal evaluation, we were encouraged by our initial results. The proof-of-concept, despite a few false positives, correctly recognized the proper category of single-and multi-word phrases in uncorrected ASR output. The more comprehensive system captures and transcribes speech and stores alternative phrase interpretations in an XML-based format used by a text-engineering framework. It does not yet use the framework to perform the language processing present in the proof-of-concept.

**Conclusion:**

The work here encouraged us that the goal is reachable, so we conclude with proposed next steps.

Some challenging steps include acquiring a corpus of doctor-patient conversations, exploring a workable microphone setup, performing user interface research, and developing a multi-speaker version of our tools.

## Background

Capturing accurate structured primary data from clinical encounters is critical to the integrity of medical practice. Without accurate data patients cannot be properly treated, and without structured data computer tools such as decision support systems cannot parse that data and assist physicians.

Furthermore, research in translational medicine also depends on our ability to document patients' clinical conditions so that we can relate these to the enormous new data sets that we can gather about patients' genes. Unfortunately, many studies document deficiencies in the record-keeping process as currently practiced by clinicians. Early studies show that actual medical records often fail to include critical information. A 1971 Army study reported critical missing data from the medical record in 10-70% of cases [1]. A 1975 study compared 51 tape-recorded conversations between patients and physicians in a pediatric clinic and found significant omissions in the record even though they had been mentioned in the recorded discussion: 6% of the reasons for the visit, 10% of the degrees of disability, 12% of allergies, 22% of compliance data, 31% of indications for follow-up and 51% of the causes of illness had been stated but not recorded [2]. More recent studies of a similar sort demonstrate that the problem persists to today's generation of physicians. For example, a 1997 study found significant disagreement between physicians' logbooks and their patients' self-report of their medical conditions, and a 1998 study found medical students underreported patient encounters by 17.3% [3,4]. Although current practice recommends the adoption of computerized records and computerized physician order entry [5], these trends are met with resistance in part because they take additional time from the practice of already busy doctors [6].

Furthermore, computerized order entry alone does not guarantee complete records. In one study, computer records were less complete than paper records in all but diagnoses, prescriptions, and referrals [7].

We explore the intriguing possibility that we can bring technology to bear on the problem of accurately capturing machine-interpretable data from the clinical encounter using a combination of automated speech recognition (ASR) systems and tools for extraction of clinical meaning from narrative medical text. Our goal is to instrument the locale of a clinical encounter (such as a doctor's office or examination room) with one or more microphones that listen to two-party conversations, transcribe them using ASR technology, annotate them using medical natural language processing (MNLP) tools, and then integrate the data they have extracted into a displayed evolving structured encounter note that is visible to both physician and patient and that can be edited by them using a natural speech and pointing interface to correct errors and complete the record.

Other researchers have mounted efforts to capture accurate patient records more automatically through technology (e.g. [8,9]), but our research is timely and novel for a number of reasons. For one, most serious efforts in this area occurred prior to this last decade's major improvements in ASR and MNLP (e.g. [8,10]), and so the technologies available to those researchers were inadequate to the task. Second, we believe we are suggesting a new approach - one that will utilize conversational interaction in an office visit and will enable the patient and provider to interact with the system during the encounter. Third, most research in ASR has focused on transcribing speech (e.g. [11]); however, we propose to use ASR in an entirely different way. Rather than capturing a simple transcript, we are using MNLP techniques to extract a structured and coded encounter summary.

**Figure 1:**
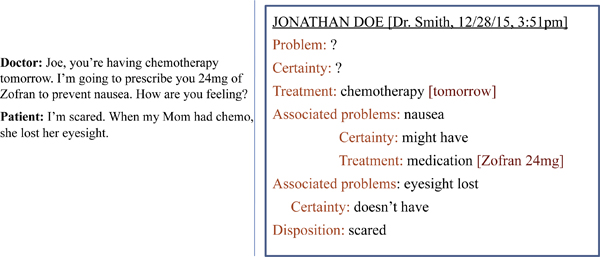
**Hypothetical encounter**. A hypothetical conversation snippet between a doctor and patient, and what the resulting encounter note display might look like.

The text of a hypothetical doctor-patient encounter and an example of what information might be displayed can be seen in Figure 1. Such a system would utilize MNLP to extract concepts from the text, classify the relationship of concepts to each other, and identify the nature of the assertions about the concept (e.g., was it part of a question, a negative statement, or a positive statement?). It would combine this information, structured hierarchically, into the encounter summary. The data in the system would flow in a bidirectional pipeline between speaker recognizer, speech recognizer, MNLP, and visual rendering. Therefore the note would evolve as the encounter progressed (forward flow), and the system would learn as corrections were made (backward flow).

This is a very ambitious goal, and so far we have taken only the most preliminary steps toward its fulfillment. Here we report a simple proof-of-concept system and the more comprehensive one we are building. The proof-of-concept permits a lash-up of Dragon's well-known Naturally Speaking ASR system [12] with an MNLP system called CaRE (Category and Relationship Extractor) built in our laboratory by Sibanda [13]. These two components allow us to experiment with recording at least one side of a conversation, finding clinically significant terms in the recognized speech, and summarizing them in a draft of the encounter note. The more comprehensive system uses GATE, the General Architecture for Text Engineering [14], to more thoroughly integrate the different components of this task.

## Methods

We first developed a simple proof-of-concept system to recognize clinically significant concept terms from a single speaker in a physician-patient encounter. Without a formal evaluation, we noted that this system was able to make a reasonable interpretation of uncorrected ASR output in a single-speaker environment. This demonstrated sufficient feasibility to move forward, so we embarked on a more comprehensive design that could support the advanced features needed in the eventual system. Such features must include multi-speaker ASR and utilization of further MNLP techniques to not only recognize concepts but also fully interpret a two-party conversation. To this end, we developed an intelligent listening framework (ILF), which is a step toward our long-term goal of a system that will capture all the relevant data from a doctor-patient encounter into a well-structured encounter note. Here we describe the two systems.

In both, we chose to use one of the most successful commercial ASR systems available, Dragon's Naturally Speaking (DNS), for interpretation of speech inputs. Colleagues at Nuance, which produces and markets DNS, have made available to us a well-documented Software Development Kit (SDK) for DNS that has allowed us to integrate its capabilities with other programs. They have also given us use of several copies of their Medical Edition, which is widely used as a transcription tool for doctors and has demonstrated good accuracy on medical speech [15].

### Proof-of-concept

**Figure 2:**
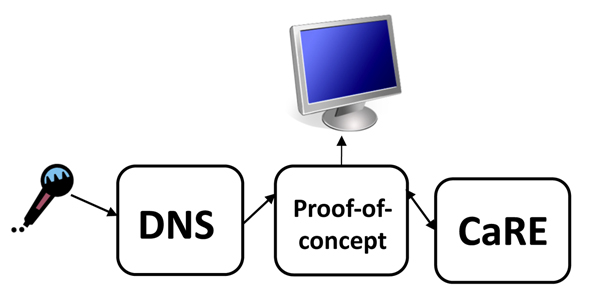
**Architecture: proof-of-concept**. A diagram of the architecture of our proof-of-concept system, showing the relationships between DNS, CaRE, and the graphical display.

Our proof-of-concept system consists of a Java program that presents a text window (hidden from the user) to DNS into which it can type, much as it normally does when used for simple dictation. At the time of its construction, we did not yet have access to the DNS SDK, hence we adopted this more straightforward, if awkward, approach. This program observes this input window and, when enough input has been gathered, invokes CaRE to try to interpret those data. It then presents the interpretation in a second window, highlighting words and terms that have been identified as clinically important ones, and showing by color highlighting the semantic types of the recognized terms. The architecture of the proof-of-concept is shown in Figure 2.

CaRE was implemented in a combination of Java and Perl programs that also invoke a number of large pre-packaged utilities such as a Support Vector Machine (SVM) based learning system [16,17], the Brill tagger [18] that uses statistical models to identify the likely parts of speech of words, and the Link Grammar Parser [19] that determines the syntactic structure of sentences and sentence fragments. It also includes custom programs that make use of a local copy of the UMLS metathesaurus [20] to identify the semantic types of words and phrases found in the text. Sibanda evaluated the performance of CaRE using the F-measure, which is the harmonic mean of precision and recall, which in turn are respectively measures of accuracy and completeness in information retrieval [21]. Applied to text from hospital discharge summaries, CaRE achieved an F-measure above 90% for retrieval of relevant medical concepts [13]. Sibanda also describes a component that recognizes relationships among words and phrases, but we have not yet exploited this capability.

Though the simple approach taken here was sufficient to persuade us that the larger task was feasible, its architecture is clearly not sufficient to handle the many additional interactive components that will be needed for the overall project. Therefore we developed the Intelligent Listening Framework (ILF).

### Intelligent listening framework

ILF is implemented as a Java program, running in Microsoft Windows, that uses the DNS SDK to control the background operation of DNS and also control GATE to create documents from the outputs of the speech interpretation process. ILF is built as a flexible tool with adjustable parameters to control interactions between DNS and GATE. For example, ILF has an adjustable granularity parameter that controls how often recognized text is sent to an MNLP package for processing and how often the recording of actual raw speech is sent (dumped) to disk. This attempts to minimize a flaw in the DNS SDK: the raw speech dump must not be done continuously, because dumping to disk always inserts a sentence break. This is because DNS normally uses its interpretation of the beginning of the next utterance to decide whether the pause that caused it to recognize two utterances does or does not correspond to an actual sentence break. The speech dump triggers DNS to reset its internal buffer, making it impossible to rely on future utterances for sentence-break information.

GATE, from Sheffield University, UK, is itself a large, Java-based integrated toolkit. GATE is distributed with a number of its own language processing tools, such as part-of-speech taggers, gazetteers and an interface to the Weka machine learning tools [14], plus it contains useful facilities for keeping track of corpora, multiple annotations, and interactive visualization and mark-up of text.

**Figure 3:**
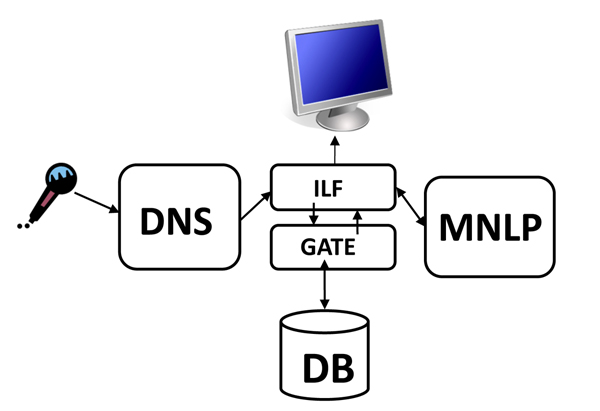
**ILF Architecture**. A diagram of the architecture of our Intelligent Listening Framework, showing the relationships between DNS, GATE, the database, and the graphical display.

We plan to re-create the MNLP processing capabilities of CaRE within the GATE framework, to allow us to experiment with variations that combine different methods for accomplishing its tasks. ILF allows more complex interfacing with MNLP than our original prototype. For example, the MNLP framework can report to ILF that it does not have enough unprocessed text to accurately interpret the speech and can request that more be captured. Because we have not yet completed the planned MNLP package of CaRE-like processing modules, we currently use a dummy processing module that simply accepts its inputs without altering or further annotating them. The ILF architecture is diagrammed in Figure 3.

Two of the principal advantages of using the DNS SDK rather than the simple dictation-to-text interface of our initial effort are that (1) the SDK can capture the actual input sounds into a raw speech file, and (2) we can query it for alternative interpretations of any portion of the speech signal. When the actual sounds being interpreted are dumped into a file by DNS, it can be instructed also to record the start and stop times of each *utterance *(these are the segments of speech between natural breaks such as pauses) and of each *word *(the units identified by the ASR algorithm). In addition, the SDK can provide a list of the top alternative interpretations of the last utterance and its confidence score for each word in the top choice. Therefore it will be possible, in a future, more integrated system, to go back and reinterpret segments of the speech input if what was transcribed does not appear to make sense. It should also be possible to build recognizers for non-speech noise sources that may occur often in our target clinical setting, such as a cough or a baby crying. With the recorded timing information for each element of the interpreted text, such a recognizer could identify segments of input that should be omitted from interpretation. Another, yet more powerful possible design that is not supported by the current SDK would permit ILF to provide feedback to the DNS recognition algorithm based on the semantic plausibility of what is being recognized.

#### Software design

The data-flow of the ILF is defined according to the following pseudocode. 'Utterance-limit' and 'dump-limit' correspond to the two granularity parameters described above. The former controls how frequently raw speech is sent to disk, and the latter controls how frequently the speech already sent to disk is processed by GATE.

Create 'unprocessed' and 'processed' GATE documents.

When DNS signals an utterance is completed:

      Move the transcript of that utterance into 'unprocessed'.

      Store alternative interpretations as GATE annotations.

After 'utterance-limit' utterances:

   Dump the speech file to disk.

   Create GATE annotations of speech file metadata (start and stop times of each word).

After 'dump-limit' speech-file dumps:

   Execute MNLP processing on 'unprocessed'.

   If MNLP completes successfully:

      Move the contents of 'unprocessed' to 'processed' and update the database.

   Else:

      Leave all information in 'unprocessed' until more speech transcript is captured.

## Results and discussion

### Proof-of-concept

Without a formal evaluation of this system, we were encouraged by our initial results. We noted that it was able to make a reasonable interpretation of uncorrected ASR output. Although there were some false positives, many concepts are correctly recognized with the proper category, including some multi-word phrases that are not built into the UMLS. Because CaRE was trained on text from discharge summaries rather than doctor-patient conversations, we also believe that its performance can be improved significantly once we train it on appropriate corpora (which we do not yet have).

**Figure 4:**
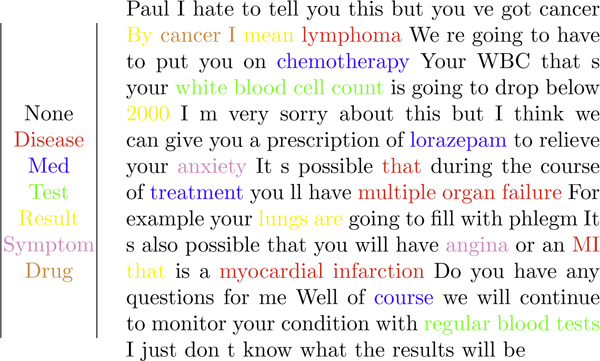
**Proof-of-concept output**. A sample run of our proof-of-concept system, showing the marked-up text (right) and the key for the mark-up (left). Notice that despite some false positives, many words and phrases are detected properly. The F-measure of detection accuracy is 73.6%. Additionally, 85.7% of the correctly identified items are also categorized correctly. Note that this is not meant to be an example of actual physician speech, but a combination of technical and non-technical medical terms in casual speech.

Figure 4 shows an example of spoken text from a one-sided conversation interpreted by this system, annotated with recognized concepts. The F-measure of detection accuracy in this example is 73.6%. Additionally, 85.7% of the correctly identified items are also categorized correctly. Note that this example is not meant to reflect the actual tone of a physician speaking to a patient. Rather, it is a demonstration of the system's ability to recognize both technical clinical concepts (e.g., myocardial infarction) and non-technical clinical concepts (e.g., regular blood tests) in the midst of casual speech.

### Intelligent listening framework

Because the MNLP processing modules were not complete at the time of this writing, we report here the ability of the system to successfully capture and transmit the output and annotations of the DNS SDK to GATE.

**Figure 5:**
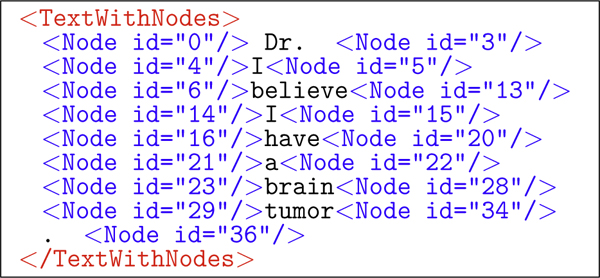
**ILF: dictated utterance**. A dictated utterance with embedded GATE annotation nodes, as XML.

**Figure 6:**
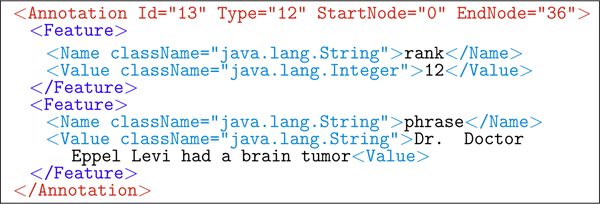
**ILF: phrase choice annotation**. The 12th-choice alternative phrase for the utterance in Figure 5.

**Figure 7:**
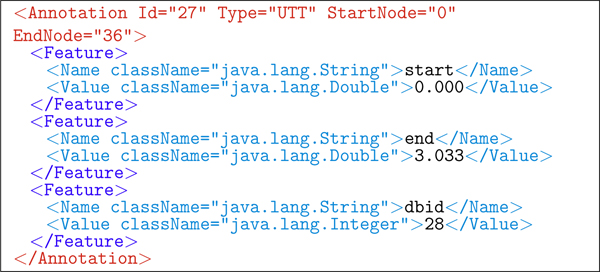
**ILF: sound-dump annotation**. The sound-dump-timing and associated WAV-file id for the utterance in Figure 5.

An example of annotated XML of a single utterance is shown in Figure 5. Figures 6 and 7 show the two types of annotations ILF automatically produces after DNS interpretation. Figure 6 is the 12th-choice alternative interpretation, and Figure 7 is the sound dump annotation, capturing both the length of the utterance and its file id on disk.

#### Evaluation plan

In the future, our laboratory will undertake a formal evaluation of the speech-recognition and the forthcoming MNLP modules. To accomplish this, we must first acquire a collection of doctor-patient conversations, which will be done by recording conversations from a toxicology clinic in the Boston area. We will develop a corpus of manual (and therefore correct) transcriptions of the recorded conversations, and we will annotate those with data elements identified from initial-encounter forms used by the toxicologists in that clinic. We can then measure the performance of transcription of the recordings against the corpus, cross-validate the MNLP modules against the corpus, and do a combined analysis in which the same partition used for MNLP-module testing is also used to evaluate transcription performance.

### Difficulties encountered

As with much of contemporary software engineering, the greatest challenges in building these tools has been to deal with the many incompatible components. Some of the difficulties we have encountered will illustrate this theme:

First, CaRE consists of a complex set of interrelated tools developed in various programming languages and originally deployed in a Linux environment, and it depends on Unix facilities to tie its pieces together. DNS runs only in Microsoft Windows and is strongly coupled to such Windows-only technologies as ActiveX controls and COM objects. GATE has been developed on a Java platform, which does not support ActiveX and COM. Consequently, integrating all of these pieces required considerable effort. To use ActiveX and COM objects, we used a Java-COM bridge. We settled on JACOB (JAva-COm Bridge) [22], which in our evaluation was the most stable and functional of the open source tools. (We also considered Jawin [23], but it does not adequately support event handling; and J-Interop [24], which is based on Distributed COM, the use of which is severely limited by Microsoft to prevent network control of Windows systems.) To allow CaRE to run on Windows, we had to find workarounds for the missing Unix facilities that tie its pieces together. For future extensions, we will almost certainly need to recode much of CaRE in Java, to allow its proper integration into the GATE framework.

Second, GATE does have an application programming interface (API), but our impression is that those who use the API are principally those already connected with the system's development. Although we find the graphical user interface presented by that system to be robust and relatively easy to use, we have had the opposite experience with the API. We found places where its behavior is not predictable from the documentation, and others where documented calls simply do not work. One particular area where we encountered serious problems has been in utilizing GATE's persistence tools, which should work best with an actual database backend. GATE describes support for the free PostgreSQL database, but due to GATE's poor support of the latest release and Windows' poor support of the prior release, we struggled to connect GATE and PostgreSQL. Eventually, we were able to implement the ILF using the GATE API and PostgreSQL. However, we continued to encounter erratic database corruption due to the unreliability of GATE's database-backed persistent document implementation. It appears that some undiscovered error in its implementation causes changes sometimes not to be communicated to the database record.

### Challenges and next steps

The current version of ILF is quite functional for the two tasks described here: (1) capturing transcribed speech and metadata from a single speaker and (2) inputting these into a database-backed text engineering framework. We have mentioned the need to incorporate into GATE the CaRE-like abilities to interpret the transcribed text into a concise and valid structured record of the encounter. This should be a "mere matter of programming," because we have previously built similar systems (e.g. [13,25], and [26]). We believe, however, that there are several other major challenges facing us in our work on this project.

First, current ASR systems seem built for use by a single user, not the pair (at least) that participate in a clinical encounter. Thus DNS expects that every utterance heard by it comes from the same speaker, hence it applies the same language and speaker model to all inputs. This is clearly wrong in our setting, and will lead to degraded performance if, for example, one party to the conversation is female and the other male, or if one has a very different accent than the other, making any language model a poor fit for both. There are, in the research laboratory, ASR systems designed to be far more speaker independent than DNS, and perhaps they could be adapted to our task. We have also considered running two instances of DNS, one listening to the doctor, the other the patient. It is not currently possible to run more than one instance of the software on a single machine, which is a shame in the era where two, four and even eight-core personal computers are becoming commonplace. Thus our current plan is to use two computers to interpret the two participants' inputs, and then to use a network-based coordination protocol to assure synchrony between what is said by the two parties. A practical impediment to this approach is the difficulty of placing two computers, rather than one, into an already crowded clincial space.

Second, although doctors may be willing to train a system to their voice patterns and speaking styles, patients certainly will not have the opportunity or time to do so. DNS does come with a generic language model that claims to be able to handle the ASR task without any training, but such use clearly degrades accuracy. Again, we may need to use more research-stage systems that have been designed explicitly for such audiences if the DNS models are inadequate.

Third, the quality and placement of microphones seems to be critical to good ASR performance. Indeed, DNS recommends use of a headset microphone, which may be suitable for dictation but is probably not acceptable in a clinical encounter. We have used such microphones in our experiments so far. Alternatives include high-quality lapel microphones, whose placement farther from the speaker's mouth puts them at a disadvantage, but which may be sufficiently unobtrusive to be acceptable. A better option would be an array microphone, which uses dynamic signal processing techniques with an array of microphone inputs, typically arranged in a line, to isolate sounds that come from a specific direction and distance in the space before them. These can be used several feet from the speaker, and thus do not interfere with the speaker's freedom of movement. Such systems had been quite expensive, but continued price reductions have now made them available for under $100. Unfortunately, our very limited experience with one such system suggests that they do not perform as well for ASR as the headset-mounted microphones.

Fourth, we must accumulate a significant set of doctor-patient conversations to use as training data in developing the statistical models that go into CaRE and similar systems. In addition, if we find that our initial serial approach to the interpretation task does not yield sufficient accuracy, we may need to develop a more sophisticated integration between various components of ILF so that quality measurements in different parts of the system can control the effort expended by other parts to reach some globally optimal interpretation.

Finally, the challenge of creating a primarily speech-based interface that will allow a doctor and patient to correct a visually-presented encounter record seems daunting. Clearly, dictation-oriented commands such as "delete last paragraph" are completely inappropriate to this setting. Instead, such corrections need to be made through natural speech, based on the semantics of what the system believes and shows. Thus, we should expect statements more like "no, he suffered his heart attack in 1985, not 1995." We are unaware of existing techniques for doing this, which raises both the risks and rewards of our approach.

## Conclusion

This first pass at our ambitious goal of automating documentation of clinical encounters yielded two positive results. First, it encouraged us that, given enough time and effort, the goal is reachable. Second, it gave us a realistic understanding of the strengths and weaknesses of the state of the art and helped us to anticipate and plan for the challenges that lie ahead.

## List of abbreviations used

ASR: Automated Speech Recognition; API: Application Programming Interface; CaRE: Concept and Relationship Extractor; COM: Component Object Model; GATE: General Architechture for Text Engineering; DNS: Dragon Naturally Speaking; ILF: Intelligent Listening Framework; JACOB: Java-COM Bridge; MNLP: Medical Natural Language Processing; SDK: Software Development Kit; SVM: Support Vector Machine; UMLS: Unified Medical Language System; XML: Extensible Markup Language

## Competing interests

The authors declare that they have no competing interests.

## Authors' contributions

JK designed and implemented the software, performed experiments, and wrote the manuscript. PS conceived of the project, participated in design and coordination, helped draft the manuscript, and approved the final manuscript.

## References

[B1] Tufo HM, Speidel JJ (1971). Problems with medical records. Medical Care.

[B2] Zuckerman AE, Starfield B, Hochreiter C, Kovasznay B (1975). Validating the content of pediatric outpatient medical records by means of tape-recording doctor-patient encounters. Pediatrics.

[B3] Patricoski C, Shannon K, Doyle G (1998). The accuracy of patient encounter logbooks used by family medicine clerkship students. Family Medicine.

[B4] Haapanen N, Miilunpalo S, Pasanen M, Oja P, Vuori I (1997). Agreement between questionnaire data and medical records of chronic diseases in middle-aged and elderly Finnish men and women. American Journal of Epidemiology.

[B5] IOM (1997). The computer-based patient record: an essential technology for health care.

[B6] Shu K, Boyle D, Spurr C, Horsky J, Heiman H, O'Connor P, Lepore J, Bates D (2001). Comparison of time spent writing orders on paper with computerized physician order entry. Medinfo.

[B7] Pringle M, Ward P, Chilvers C (1995). Assessment of the completeness and accuracy of computer medical records in four practices committed to recording data on computer. The British Journal of General Practice.

[B8] Shiffman S, Detmer M (1995). A Continuous-speech Interface to a Decision Support System: I. Techniques to Accomodate for Misrecognized Input. J Am Med Informatics Assoc.

[B9] Lacson R, Barzilay R, Long W (2006). Automatic analysis of medical dialogue in the home hemodialysis domain: structure induction and summarization. J Biomed Inform.

[B10] Rosenthal DF, Bos JM, Sokolowski RA, Mayo JB, Quigley KA, Powell RA, Teel MM (1997). A Voice-enabled, Structured Medical Reporting System. J Am Med Inform Assoc.

[B11] Bellegarda J (1997). Statistical techniques for robust ASR: review and perspectives. EUROSPEECH-1997.

[B12] Nuance Communications (2008). Dragon Naturally Speaking. http://www.nuance.com/naturallyspeaking.

[B13] Sibanda T (2006). Was the Patient Cured?: Understanding Semantic Categories and their Relationship in Patient Records.

[B14] Cunningham H, Maynard D, Bontcheva K, Tablan V (2002). GATE: A framework and graphical development environment for robust NLP tools and applications. Association for Computational Linguistics Proceedings.

[B15] Devine EG, Gaehde SA, Curtis AC (2000). Comparative evaluation of three continuous speech recognition software packages in the generation of medical reports. JAMIA.

[B16] Cristianini N, Shawe-Taylor J (2000). An introduction to support vector machines.

[B17] Chang CC, Lin CJ (2001). LIBSVM: a library for support vector machines.

[B18] Brill E (1992). A Simple Rule-based Part of Speech Tagger.

[B19] Temperley D (1999). An Introduction to the link grammar parser. http://www.link.cs.cmu.edu/link/dict/introduction.html.

[B20] Humphreys BL, Lindberg DA (1993). The UMLS project: making the conceptual connection between users and the information they need. Bulletin of the Medical Library Association.

[B21] Bramer M (2007). Principles of Data Mining.

[B22] Adler D (2008). A JAva-COM Bridge. http://sourceforge.net/projects/jacob-project.

[B23] Martin R, Halloway S, Gehtland J (2008). Jawin - A Java/Win32 interoperability project. http://jawinproject.sourceforge.net/.

[B24] Roopchand V (2008). j-Interop: Pure Java - COM Bridge. http://j-interop.org.

[B25] Shu J (2005). Free text phrase encoding and information extraction from medical notes.

[B26] Long W (2007). Lessons extracting diseases from discharge summaries. AMIA Annual Symposium Proceedings.

